# Changes in colorectal cancer screening intention among people aged 18–49 in the United States

**DOI:** 10.1186/1471-2458-14-901

**Published:** 2014-09-01

**Authors:** Mary L Greaney, Elaine Puleo, Kim Sprunck-Harrild, Sapna Syngal, Elizabeth Gonzalez Suarez, Karen M Emmons

**Affiliations:** Health Studies & Department of Kinesiology, University of Rhode Island, 25 West Independence Way, Kingston, RI 02881 USA; University of Massachusetts, School of Public Health and Health Sciences, Amherst, MA 01003 USA; Center for Community-Based Research, Dana Farber Cancer Institute, 450 Brookline Avenue, Boston, MA 02215 USA; Dana-Farber Cancer Institute, 450 Brookline Avenue, Boston, MA 02215 USA; Brigham and Women’s Hospital, Boston, MA 02215 USA; Harvard School of Public Health, 655 Huntington Ave., Boston, MA 02215 USA

**Keywords:** Colorectal cancer, Cancer screening, Screening intentions, Low-income population

## Abstract

**Background:**

To determine whether exposure to a peer-led intervention focused on colorectal cancer (CRC) screening, physical activity, and multi-vitamin intake can lead to increased intentions to be screened for CRC once age eligible among adults under the age of 50.

**Methods:**

Participants were residents of low-income housing sites, and CRC screening intentions were assessed at baseline and at follow-up (approximately 2 years later) to determine changes in screening intentions and factors associated with changes in intentions.

**Results:**

Participants (n = 692) were 78.4% female, 42.6% Hispanic and 50.8% black. At follow-up, 51% maintained their intention to be screened and 14.6% newly intended to get screened. Individuals newly intending to get screened were more likely to have participated in the intervention, be older, male, and born in Puerto Rico or the United States compared to those who maintained their intention not to get screened (p < 0.05).

**Conclusion:**

Exposure to CRC prevention messages before the age of 50 can increase screening intentions among individuals who did not initially intend to get screened. Peer-led interventions to promote CRC screening should include individual less than 50 years of age, as this may contribute to increased screening at the recommended age threshold.

## Background

Colorectal Cancer (CRC) is highly preventable, yet is the second leading cause of cancer death in the United States [1, 2]. As early detection is associated with reduced morbidity and mortality [3], the U.S. Preventive Services Task recommends that CRC screening begin at age 50 [4]. Although screening rates are increasing among adults 50 years of age or older [5], recent analysis of data from the 2010 Behavioral Risk Factor Surveillance Survey (BRFSS) determined that only 55.1% of individuals aged 50–59 are current with CRC screening [6]. Adherence to CRC screening recommendations increased with age, with 72.9% of respondents aged 60–69 and 76.9% aged 70–75 being current with CRC screening [6]. Data from the National Health Interview Survey confirm that CRC screening rates are lower among younger respondents (aged 50–64) than among older respondents (aged 65+) [7, 8]. Combined, these data clearly indicate that screening rates need to increase among younger populations eligible for CRC screening (e.g., individuals ages 50–59), as a delay in screening may prevent early detection, which is critical to prognosis. Early detection positively tracks survival rates [9], and increasing the number of people who get screened regularly, starting at age eligibility, which in the United States is 50 years of age, may increase early detection, thereby reducing avoidable mortality. Furthermore, screening rates in all age groups are notably lower among racial/ethnic minority groups [1, 10–12], lower income groups [1, 11, 12], the uninsured [11, 13, 14], those born outside of the US [15], and individuals with less educational attainment [7, 12, 14]. Therefore, increasing screening among these populations when they first become age eligible has the potential to reduce existing disparities in CRC-related morbidity and mortality.

Intention to perform a behavior is one of the strongest immediate determinants of behavior [16]. Intention to participate in cancer screening is associated with completed cancer screening [17–19], as well as return for routine annual cancer screening [20]. We previously examined CRC screening intentions among adults less than 50 years of age living in low-income housing sites who participated in the Open Doors to Health study (ODH); 66% of participants intended to be screened once age-eligible. Individuals with more role responsibilities (e.g. child or parental care giving and primary economic obligation), greater role conflicts (e.g. conflict between daily activities and life demands), and reported higher levels of social cohesion in their community had a greater intention to participate in CRC screening upon turning 50 [21]. Identifying factors associated with changes in CRC screening intentions among adults less than 50 may allow for salient programs and communication strategies to be developed, which may contribute to an increase in the number of adults who participate in CRC screening as soon as they turn 50 and potentially reduce preventable morbidity and mortality. Thus, this current study was conducted to: 1) determine if the ODH intervention led to changes in screening intentions and 2) examine factors associated with a change in CRC screening intentions among ODH participants aged 18–49.

## Methods

### Study design

This paper is a secondary analysis of data from ODH, which has been discussed in detail elsewhere [21–23]. Briefly, ODH was a CRC prevention trial delivered within 12 low-income housing sites in metropolitan Boston, Massachusetts, United States. Low-income housing sites are an important venue for promoting healthful behaviors, including cancer screening [24].

ODH employed a cluster-randomized design with housing sites as the primary sampling unit (six housing sites were intervention sites, six were control sites), and the participant was the secondary sampling unit. This study design was used as the intervention was delivered at the housing site. The intervention was guided by the social contextual model, which recognizes that individual, interpersonal, community, and organizational/systems-level factors influence health behaviors, and explicates the role that social contextual factors have on health behaviors across these different levels [25]. Both intervention and control sites received increased screening access.

In the intervention sites, residents interested in colonoscopy received from a peer-leader or program staff a package with user-friendly screening preparation instructions and one-to-one education about how to bring this package to the next primary care provider (PCP) appointment and how to discuss his/her desire to get screened with the PCP. In the control sites, the user-friendly instructions were mailed to each resident participating in the study. The instructions were mailed with a letter that explained how to contact a program staff to help them prepare and get a screening appointment. Study participants could be scheduled for endoscopy appointments within six weeks of referral by the patient's PCP, through a partnership with a screening program at a local hospital. The screening program provided appointment reminders, and transportation if needed. The educational materials distributed at all sites included information about fecal occult blood tests (FOBT), flexible sigmoidoscopy and colonoscopy. These tests also were discussed in the group sessions (see below) held at the intervention sites. Intervention sites also received a peer-led intervention, delivered in Spanish and English, targeting screening, physical activity, and multi-vitamin use. Two to three residents at each intervention site were recruited and trained to serve as peer leaders. Peer-led interventions have been used with success to increase rates of cancer screening and to promote healthful behaviors among low-income, ethnically diverse groups [26, 27], although, to our knowledge, no other peer-led interventions have been designed to promote CRC among residents of low-income housing sites. The peer leaders conducted ongoing outreach and follow-up with residents. They also served as co-leaders with research staff on intervention activities or oversaw intervention activities, which included: 1) community events (i.e., health fairs); 2) group sessions about the intervention targets; 3) monthly poster campaigns featuring intervention targets; 4) resource boards in common spaces that were updated quarterly; 5) ongoing outreach and follow-up; and 6) weekly on-site physical activity series and walking clubs for four 8-week periods. In both intervention and control sites, residents were offered equivalent access to screening through expedited access to endoscopy appointments at two local hospitals for those choosing endoscopy (vs. home test kits).

Participant eligibility requirements included: 1) being a resident of a participating housing site; 2) being 18+ years of age; 3) being fluent in English or Spanish; and 4) not undergoing cancer treatment. Housing site representatives sent eligible residents a letter that introduced the study, and study staff initiated follow-up contact via telephone or home visits to determine interest. All participants provided informed consent and completed the interviewer administered baseline and follow-up surveys in English or Spanish. Baseline data were collected between 2004 and 2005; and the follow-up survey was administered from September 2006 to January 2008. The response rate for the follow-up survey was 81%. The ODH study was approved by the Human Subjects Committee at the Harvard School of Public Health. The present study is limited to ODH participants who at baseline had not been screened for CRC and were between 18 and 49 years of age and who completed the baseline and follow-up surveys.

### Measures

*CRC screening intention* was assessed at the baseline and follow-up survey by the question: “Do you plan to be screened for colon cancer?” (yes, no, don’t know) [21]. These data were used to create the variable, change in CRC screening intention. To do this, we first recoded all “don’t know” responses (n = 63 at baseline, n = 18 at follow-up) on both surveys as “no”, and classified individuals who reported being screened for CRC at follow-up as intending to get screened. We created four categories reflecting change in CRC screening intention: 1) consistent positive intention (yes at baseline and follow-up), 2) new intention (no at baseline, yes at follow-up), 3) discontinued intention (yes at baseline, no at follow-up), and 4) consistent negative intention (no at baseline and follow-up).

#### Contextual factors

We measured role responsibilities, role conflicts, and social cohesion. *Role responsibilities* were assessed by querying participants about how much responsibility they had for earning money to support the family and taking care of children and/or their household. Response options included *little or none, about half*, and *most or all*. Role responsibilities was computed as the number of family roles for which the participant was mostly or fully responsible (0 to 3) and this was used to create a dichotomous variable (0–1, 2–3) [28]. *Role conflicts* were assessed by asking participants whether their daily activities created conflicting demands in their life (yes, no) [28]. *Social cohesion* was measured by 5 items that assess perceptions of trust and shared values in one’s neighborhood. A summary score was calculated (range: 0–4) with a higher score indicating greater levels of social cohesion than a lower score [29].

#### Health care factors

Participants reported if they had a regular doctor or nurse practitioner (NP) and how many times they had seen that clinician in the past year. They also reported how well their provider understands their social context by answering how well their doctor/NP knows: 1) their responsibilities at work, home, or school; 2) their worries about their health; and 3) them as a person, and their values and beliefs. Responses included *not at all*, *a little*, *somewhat*, and *very well.* One point was given for each question for which the participants answered *somewhat* or *very well,* and a summary score was created [30]. We also determined insurance status (private, public, private + public, uninsured).

#### Socio-demographic characteristics

Participants reported sex, age, race/ethnicity, education, whether English was their primary language, and place of birth, which was collapsed to three categories (US, Puerto Rico, Other). We determined yearly household income (<$10,000, $10,000-$19999, $20,000-$29,999, $30,000-$39,999, $40,000-$49,999, or $50,000+) and the number of individuals supported on this income. Thus information was used to determine if participant’s household income was being at/above or below poverty line based on the 2005 federal poverty guidelines for income and household size [31].

### Analysis

Due to the cluster design, data were weighted up to the population size within each housing site (n = 692, weighted sample = 1,004). We calculated descriptives for the key variables, and conducted bivariate analyses to examine the associations between the independent variables and change in CRC screening intentions. Using an intention to treat approach, variables that were significant at p < .10 overall in bivariate analyses in one or more models were initially entered into a series of cluster randomized, multivariable logistic regression models in addition to intervention status, age, and gender which were selected *a priori*. Next for each individual intention initial multivariable model, covariates with the highest Wald p-value were removed one at time to improve model fit, until all variables left in model were significant other than intervention status, age, and gender which were selected *a priori*. Next for each individual intention initial multivariable model, covariates with the highest Wald p-value were removed one at time to improve model fit, until all variables left in model were significant other than intervention status, age, and gender which were selected *a priori*. The referent group for all logistic regression models was consistent negative intention. Analyses were conducted using SUDAAN 9.01 and SAS 9.3 statistical software.

## Results

**Figure 1:**
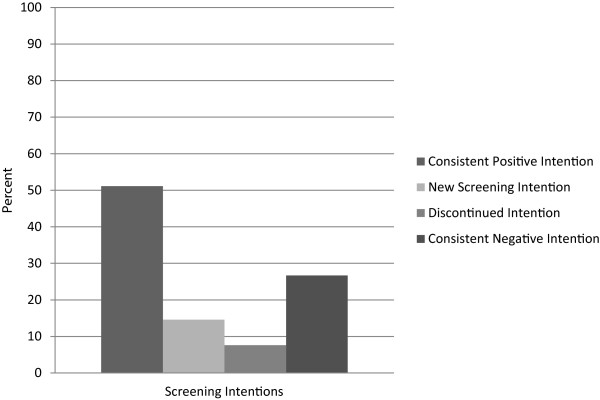
**Longitudinal changes over 2 years in colorectal cancer screening intentions (weighted sample n = 1004).**

Participants were predominately female, non-White and 42.3% lived at or below the federal poverty line; the sample had a mean age of 34.3 (SE = 0.3) years (see Table 1). Over half (51.1%, weighted sample n = 513) of participants maintained a consistent positive screening intention and 14.6% (weighted sample n = 147) had a new screening intention. However, 7.6% (weighted sample n = 76) of participants who reported at baseline that they intended to get screened when age eligible no longer had this intention at follow-up and 26.7% (weighted sample n = 268) had a consistent negative screening intention (see Figure 1).

**Table 1:** **Weighted baseline characteristics of study samples (n = 1,004)**

Socio-demographics	N (unweighted sample)	% or Mean (SE) ^a^
Age (SE)	692	34.3 (0.3)
Female	535	78.4
Race/ethnicity		
Hispanic	277	42.6
Black	372	50.8
White/Other	41	6.6
Poverty line^b^		
Above poverty line	313	45.2
At or below poverty line	293	42.3
Missing	86	12.4
Education		
< high school (HS) diploma	142	21.8
HS diploma or equivalent	227	32.1
More than HS diploma	322	46.1
Place of birth		
US	422	59.2
Puerto Rico	107	17.3
Other	162	23.4
% English 1^st^ language	401	55.6
Insurance status		
None	40	5.9
Public only	367	53.1
Private only	229	33.1
Public + private	52	8.0
**Socio-contextual factors**		
Social cohesion (mean)	690	2.49 (0.03)
# Role responsibilities^b^		
0-1	260	37.9
2-3	408	62.1
% Have role conflicts	278	42.7
**Health care factors**		
% Have regular MD/NP	544	80.5
MD/NP^c^ understands social context		
Not at all	250	36.0
A little	75	12.2
Somewhat	102	15.7
Very well	241	36.1
Number of times saw regular MD/NP last year		
0	74	10.6
1-3	347	49.2
4 < 12	202	30.3
12+	67	9.9

Note: ^a^The percent or mean (SE) are from the weighted sample. ^b^Number of role responsibilities is the number of roles (earning money to support the family; taking care of children, taking care of household) for which the participant had most or all the responsibility. ^C^NP = nurse practitioner.

As shown in Table 2, variables age, gender, place of birth, English as a 1^st^ language, social cohesion, role responsibilities, and role conflicts were significant at p < 0.10 in one or more of the bivariate analyses. Compared with individuals with a consistent negative intention, participants newly intending to get screened were more likely to have been in the intervention arm, be older (thus closer to the recommended screening age), and to have been born in the US. Similarly, individuals with consistent positive screening intention were more likely to be older, male, and born in Puerto Rico or the US than those with a consistent negative intention. Participants with a discontinued screening intention had fewer role responsibilities than participants with a consistent negative intention. Individuals in the intervention arm were 63% more likely to have new screening intention than those in the control arm.

**Table 2:** **The bivariate associations and the final multivariate models predicting change in colorectal cancer (CRC) screening intention (v. consistent negative intention)** ^**a**^ **among study participants (sample n = 692, weighted sample = 1,004)**

	Change in CRC screening Intentions					
	Consistent positive intention ^***b***^		New intention ^***c***^		Discontinued intention ^***d***^	
	Bivariate associations	Final Model	Bivariate associations	Final model	Bivariate associations	Final model
	OR (95% CI)	OR (95% CI)	OR (95% CI)	OR (95% CI)	OR (95% CI)	OR (95% CI)
**Intervention Status**						
Intervention (v. control)	1.38 (0.65, 2.92)	1.52 (0.73, 3.16)	1.31 (0.81, 2.12)	1.63 (1.03, 2.57)**	0.82 (0.25, 2.76)	0.91 (0.20, 4.05)
**Socio-demographics**						
Age (SE)	1.03 (1.01, 1.06)**	1.04 (1.02, 1.07)**	1.02 (1.00, 1.05)*	1.04 (1.01, 1.07)**	1.02 (1.00, 1.05)*	1.02 (0.98, 1.06)
Male (v. female)	1.89 (1.05. 3.43)**	2.28 (1.06, 4.09)**	1.65 (0.58, 4.74)	2.05 (0.64, 6.61)	1.80 (0.39, 8.21)	1.90 (0.38, 9.45)
Race/ethnicity						
Hispanic (v. White/Other)	0.83 (0.39, 1.79)		0.76 (0.29, 2.00)		1.30 (0.38, 4.48)	
Black (v. White/Other)	0.70 (0.27, 1.77)		0.91 (0.34, 2.45)		0.57 (0.14, 2.32)	
Below/At poverty line (v. above)	1.26 (0.77, 2.06)		1.38 (0.76, 2.52)		1.47 (0.73, 2.98)	
Education						
< high school diploma (HSD) (v. > HSD)	1.23 (0.85, 1.77)		1.30 (0.86, 1.98)		0.91 (0.40, 2.03)	
HSD or equivalent (v. > HSD)	1.23 (0.72, 2.07)		1.50 (0.77, 2.93)		1.11 (0.37, 3.36)	
Place of birth						
US	1.51 (0.79, 2.86)	1.94 (1.06, 3.53)**	3.04 (1.84, 5.02)**	3.71 (2.21, 6.23)**	1.48 (0.58, 3.77)	
Puerto Rico	1.63 (1.10, 2.42)**	1.54 (1.07, 2.21)**	2.10 (0.98, 4.49)*	1.99 (0.84, 4.68)	1.36, (0.58, 3.19)	
Other	REF	REF	REF	REF	REF	
English 1^st^ language (Yes v. no)	1.38 (0.85, 2.24)		1.89 (1.09, 3.28)**		1.09 (0.53, 2.27)	
Insurance status						
None (v. Public + private)	1.04 (0.27, 3.92)		1.73 (0.34, 8.87)		0.87 (0.20, 3.88)	
Public only (v. Public + private)	0.97 (0.35, 2.70)		2.25 (0.46, 10.90)		0.90 (0.28, 2.92)	
Private only (v. Public + private)	1.21 (0.48, 3.06)		1.87 (0.45, 7.72)		0.78 (0.23, 2.68)	
**Contextual factors**						
Social cohesion (mean)	1.32 (0.94, 1.87)		1.30 (0.78, 2.17)		0.98 (0.47, 2.04)	
# Role responsibilities (0-1 v. 2–3)	0.64 (0.41, 1.00)**		0.91 (0.45, 1.83)		0.47 (0.27, 0.84)**	0.49 (0.27, 0.90)**
Roles conflicts (yes v. no)^e^	1.21 (0.79, 1.87)		0.61 (0.30, 1.25)		1.86 (0.92, 3.72)*	
**Health care factors**						
Have regular MD or NP^e^ (yes v. no)	1.05 (0.62, 1.75)		0.71 (0.43, 1.17)		0.69 (0.42, 1.14)	
MDR/NP understands social context						
0 (v. 3)	0.63 (0.40, 0.98)**		0.99 (0.54. 1.79)		0.98 (0.39, 2.47)	
1 (v. 3)	0.88 (0.40, 1.90)		1.44 (0.29, 7.24)		1.62 (0.57, 4.62)	
2 (v. 3)	1.08 (0.70, 1.65)		0.88 (0.46. 1.69)		0.61 (0.13, 2.78)	
# times saw regular MD/NP last year						
0 (v. 12+)	0.65 (0.27, 1.55)		1.30 (0.34, 4.95)		0.87 (0.12, 6.32)	
1-3 (v. 12+)	0.93 (0.49, 1.78)		0.81 (0.26, 2.50)		1.11 (0.12, 10.16)	
4 < 12 (v. 12+)	1.14 (0.59, 2.19)		0.94 (0.32, 2.76)		1.14 (0.11, 11.79)	

Notes: ^a^For all models “consistent negative intention [no screening intention at baseline and follow-up]” is the referent; ^b^Continued positive intention (yes at baseline and follow-up); ^c^New screening intention (no at baseline , yes at follow-up); ^d^Discontinued intention (yes at baseline, no at follow-up); ^e^ OR = odds ratio; ^f^ NP = nurse practitioner; ^e^Number of role responsibilities is the number of roles (earning money to support the family; taking care of children, taking care of household) for which the participant had most or all the responsibility. *significant at p = .10; **significant at p = 0.05.

## Discussion

As early detection is associated with a reduction in CRC-related morbidity and mortality, efforts to increase screening among those with the lowest uptake, including newly age-eligible individuals, racial/ethnic minorities and those with limited incomes, is critical. Thus, it is important to determine if participation in CRC prevention programs before age 50 can increase screening intentions. Study results confirm that exposure to a peer-led CRC prevention education and outreach program before the age of 50 can increase screening intentions among low-income individuals.

Nearly all participants were insured (94.2%), had a healthcare provider whom they could name (80.5%), and had seen their provider at least once in the past year (89.3%). Taken together, these factors should lead to a population inclined to get screened, yet at baseline 41.3% of participants did not intend to get screened, and our intervention increased screening intentions among these individuals. Unfortunately, however, more than one-third (34.3%) of participants (i.e., individuals with discontinued intention and consistent negative intention) did not intend to get screened at follow-up, which is very concerning. Women and individuals born outside of the US or Puerto Rico were less likely to intend to get screened once age eligible, and it is possible that this lack of intention is due to other more pressing and immediate concerns (e.g., family obligations, financial constraints) [32–35].

It is somewhat surprising that the examined health care variables were not associated with change in CRC screening intentions in the final models. Previous research among individuals over 50 has found that discussing CRC screening with a health care provider is positively associated with screening intentions [10], and that a provider’s understanding of a patient’s social context is associated CRC screening [30]. Most participants had insurance and a regular healthcare provider whom they had seen in the previous year; however, CRC screening may not have been a focus of these visits given that participants were not yet age eligible. Health professionals may want to begin discussing CRC screening with patients well before they are age-eligible for screening to increase CRC screening rates among their patients who are in their 50s.

It is also surprising that the examined contextual factors were not associated with positive changes in CRC screening intention. We had hypothesized that individuals reporting greater social cohesion would be consistent in their positive screening intentions or have a new screening intention. Prior research has found that neighborhood-level cohesion is linked to beneficial health outcomes, such as reduced mortality [36, 37] and increased/improved physical activity [38]. We also had hypothesized that participants reporting greater role responsibilities and/or role conflicts would be consistent in their negative screening intentions due to competing priorities and time constraints. However, individuals who reported fewer roles and responsibilities were more likely to have a discontinued screening intention than were individuals who remained consistent in the intention to get screened. It is possible that individuals with fewer day-to-day responsibilities and/or individuals depending on them may feel that taking care of their future health only impacts themselves and thus is not as much of a priority [39, 40].

This study should be considered in the context of its limitations and strengths. One limitation is that we did not assess the reasons participants intended or did not intend to get screened. Further, by study design, we were not able to determine screening status once participants reached age eligibility. Additionally, we can not accurately determine at what age people with positive screening intentions intended to get screened (e.g. as soon as turning 50 vs. later). It is possible that individuals who intended to get screened for CRC did not intend to be screened as soon as they turned 50. Study strengths include a longitudinal study design and sample that was racially/ethnically diverse and largely low-income, although this may limit generalizablity to populations with higher incomes. However, since, CRC screening rates are lowest among lower income groups [1, 11, 12], investigation screening uptake in this group, despite the possibility generalizability issues, has value. Study strengths include a longitudinal study design and sample that was racially/ethnically diverse and largely low-income, although this may limit generalizablity to populations with higher incomes. In addition, this is one of the first studies, to our knowledge, to examine longitudinal change in CRC screening intention among younger adults.

Future research is warranted to discern whether CRC screening education prior to the age of 50 increases uptake of CRC cancer screening upon turning 50. As well, additional research, using similar interventions, would be well poised to determine if the same level of success can be achieved among populations with less health care access, as it is possible that these interventions could have an even stronger impact on promoting positive screening intentions.

## Conclusion

This study confirms that interventions have the potential to create positive changes in screening intentions among low-income urban adults under the age of 50. Participants newly intending to get screened were more likely to have been in the intervention arm, be older, and to have been born in the US. Continuing these types of efforts are important, as increasing CRC screening among newly age eligible patients, especially populations with the greatest CRC burden, could significantly reduce CRC morbidity and mortality and address existing CRC disparities. Study results clearly support encouraging individuals less than 50 years of age to participate in peer-led interventions designed to promote CRC screening as one mechanism to increase intentions to be screened.

## Abbreviations

BRFSS: Behavioral Risk Factor Surveillance Survey
CRC: Colorectal cancer
FOBT: Fecal occult blood tests
ODH: Open Doors to Health
PCP: Primary care provider.
